# Emodin, an Emerging Mycotoxin, Induces Endoplasmic Reticulum Stress-Related Hepatotoxicity through IRE1α–XBP1 Axis in HepG2 Cells

**DOI:** 10.3390/toxins15070455

**Published:** 2023-07-12

**Authors:** Su Been Park, Gun Hee Cho, Young Eun Park, Hyang Sook Chun

**Affiliations:** School of Food Science and Technology, Chung-Ang University, Anseong 17546, Republic of Korea; sirius6100@naver.com (S.B.P.); joo2413@naver.com (G.H.C.); asd3514@cau.ac.kr (Y.E.P.)

**Keywords:** emodin, mycotoxin, ER stress, apoptosis, HepG2 cells, hepatotoxicity, IRE1α−XBP1

## Abstract

Emodin, an emerging mycotoxin, is known to be hepatotoxic, but its mechanism remains unclear. We hypothesized that emodin could induce endoplasmic reticulum (ER) stress through the inositol-requiring enzyme 1 alpha (IRE1α)–X-box-binding protein 1 (XBP1) pathway and apoptosis, which are closely correlated and contribute to hepatotoxicity. To test this hypothesis, a novel IRE1α inhibitor, STF-083010, was used. An MTT assay was used to evaluate metabolic activity, and quantitative PCR and western blotting were used to investigate the gene and protein expression of ER stress or apoptosis-related markers. Apoptosis was evaluated with flow cytometry. Results showed that emodin induced cytotoxicity in a dose-dependent manner in HepG2 cells and upregulated the expression of binding immunoglobulin protein (BiP), C/EBP homologous protein (CHOP), IRE1α, spliced XBP1, the B-cell lymphoma 2 (Bcl-2)-associated X protein (Bax)/Bcl-2 ratio, and cleaved caspase-3. Cotreatment with emodin and STF-083010 led to the downregulation of BiP and upregulation of CHOP, the Bax/Bcl-2 ratio, and cleaved caspase-3 compared with single treatment with emodin. Furthermore, the apoptosis rate was increased in a dose-dependent manner with emodin treatment. Thus, emodin induced ER stress in HepG2 cells by activating the IRE1α–XBP1 axis and induced apoptosis, indicating that emodin can cause hepatotoxicity.

## 1. Introduction

**Figure 1:**
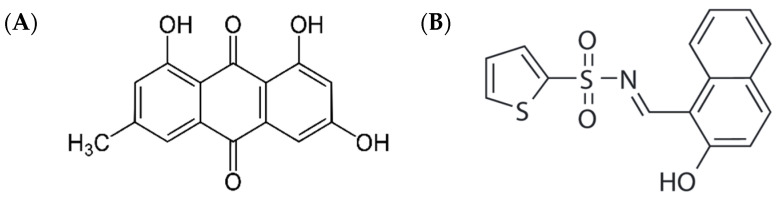
Structures of (**A**) emodin and (**B**) STF-083010, as accessed from the Sigma-Aldrich (St. Louis, MO, USA) (https://www.sigmaaldrich.com/KR/ko/product/usp/1235059 (accessed on 12 April 2023)) and Selleckchem (Houston, TX, USA) (https://www.selleckchem.com/products/stf-083010.html (accessed on 12 April 2023)) websites, respectively.

Mycotoxins are secondary metabolites produced by fungi and are commonly found in food and feed [1]. Emodin (1,3,8-trihydroxy-6-methylanthracene-9,10-dione) is an emerging mycotoxin produced by fungi from the *Aspergillus*, *Penicillium*, and *Talaromyces* genera (Figure 1A) [2,3]. By definition, an emerging mycotoxin is neither routinely determined nor legislatively regulated but whose contamination reports are increasing [3]. Emodin contamination has been reported in a wide range of foods, such as bread, fruits, vegetables, and nuts [4]. Recently, a high prevalence of emodin was reported in global finished pig feed samples (90% of 524 samples) and corn silage samples (>50% of 33 samples), respectively [5,6]. Uniquely, emodin is a compound found in plant natural products such as the dried root tuber of *Fallopia multiflora*, known as Radix Polygoni Multiflori (PMR) and is also known as one of its major components [7]. Emodin is an attractive therapeutic agent because of its anticancer, antiviral, anti-inflammatory, antibacterial, anti-allergic, antidiabetic, and other pharmacological effects [8,9]. PMR is a traditional Chinese medicine but has been demonstrated to induce toxicity, especially hepatotoxicity, under long-term use or high-dose conditions [10], yet the PMR components causing hepatotoxicity remain unclear. As a major component of PMR, emodin has been implicated in its hepatotoxicity [11].

Despite studies on hepatotoxicity induced with emodin, the underlying mechanism has not been fully elucidated [8,12,13,14]. One metabolomic study demonstrated the potential of emodin to disturb glutathione and fatty acid metabolism, supporting that emodin can induce hepatotoxicity [12]. Another study [8] reported that emodin induces apoptosis through the mitochondrial apoptosis pathway and generates reactive oxygen species (ROS) in HepaRG cells. Given that the generation of ROS is linked to endoplasmic reticulum (ER) stress [13,14], we hypothesized that emodin could induce hepatotoxicity through ER stress.

The ER is the major organelle responsible for protein/lipid synthesis, calcium storage, and signal transduction. The process that disturbs ER function is called ER stress, and the response of cells to ER stress is the unfolded protein response (UPR), which regulates the expression of target genes to maintain cell homeostasis. ER stress is a hallmark of several diseases that can occur when the stress leads to cell death or when pathological conditions impair the ability to overcome ER stress. ER stress is associated with various responses and the pathogenesis of several diseases, including inflammation, viral infection, cancer, and metabolic disease [15]. The UPR restores ER function under ER stress conditions [16]. Pro-survival signaling is converted to pro-apoptotic signaling [17]; thus, ER stress and the induction of apoptosis are closely related. Apoptosis, also known as “programmed cell death,” is characterized by several morphological features, such as cell shrinkage and chromatin fragmentation [18]. The biochemical changes in apoptosis include protein cleavage or crosslinking and DNA degradation [19]. The three main phases of ER stress-induced apoptosis are initiation, commitment, and execution. Mediators of the initiation phase are protein kinase RNA-like endoplasmic reticulum kinase (PERK), activating transcription factor 6 (ATF6), and inositol-requiring enzyme 1 (IRE1); mediators of the commitment phase are C/EBP homologous protein (CHOP), growth arrest and DNA damage-inducible protein 34 (GADD34), tribbles pseudokinase 3 (TRIB3), and the B-cell lymphoma 2 family (Bcl-2); and mediators of the execution phase are mainly caspases [17].

According to a previous study, emodin induced ER stress through the binding immunoglobulin protein (BiP)/IRE1/CHOP signaling pathway, and caused ER-related apoptosis in LO2 cells [20]. However, compounds that can be used to investigate whether emodin affects the IRE1–X-box-binding protein 1 (XBP1) signaling pathway, such as an IRE1 signaling pathway inhibitor, were not used. STF-083010 (Figure 1B), a novel IRE1–XBP1 inhibitor, inhibits XBP1 splicing by affecting only the endonuclease activity of IRE1α, not its kinase activity [21]. In addition, in a recent study, LO2 cells were identified as a derivative of the cervical cancer line HeLa, similar to Chang liver cells; therefore, caution should be exercised when interpreting the data obtained using LO2 cells [22].

In this study, the gene and protein expression levels of ER stress markers, such as BiP, IRE1α, and CHOP, and apoptosis markers, such as Bcl-2-associated X protein (Bax), Bcl-2, and cleaved caspase-3, were evaluated following treatment with different doses of emodin. Furthermore, the gene and protein expression levels of ER stress and apoptosis markers were evaluated after the cotreatment of HepG2 hepatocytes with emodin and STF-083010. Therefore, we endeavored to determine whether emodin contributes to hepatotoxicity, at least in part, through the induction of apoptosis by affecting the IRE1α−XBP1 signaling pathway.

## 2. Results

### 2.1. Decrease in the Metabolic Activity of HepG2 Cells Induced with Emodin

**Figure 2:**
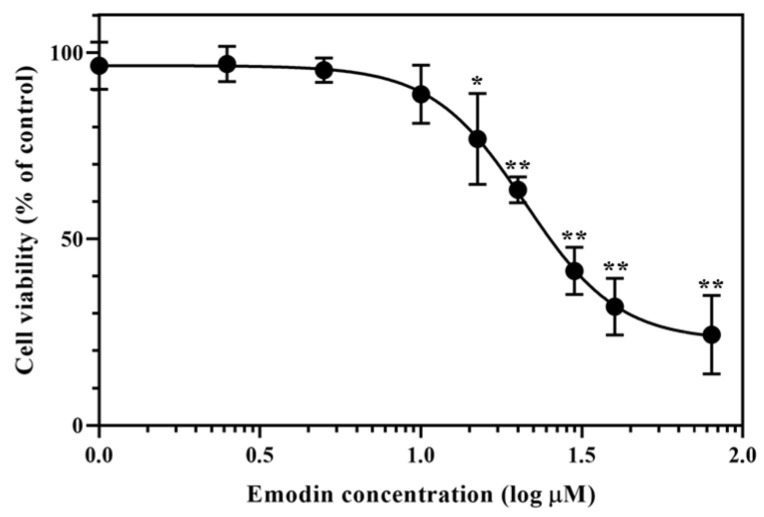
Effect of emodin on the metabolic activity of HepG2 cells. HepG2 cells were treated with emodin for 24 h, and metabolic activity was measured with an MTT assay. The IC_50_ value was determined to be 20.93 μM. Data are shown as the mean ± standard deviation (SD) from three independent experiments. * *p* < 0.05, ** *p* < 0.01 (*t*-test) compared with the vehicle control group. The statistical power was ≥0.8, which means that a non-significant result is likely to be not really significant.

To determine the cytotoxic effects of emodin on HepG2 cells, the cellular metabolic activity was measured after treatment with emodin (0–80 μM) for 24 with a 3-4,5-dimethyl-2-thiazolyl-2,5-diphenyl-2H-tetrazolium bromide (MTT) assay. Emodin treatment led to a dose-dependent decrease in cell metabolic activity, with a half maximal inhibitory concentration (IC_50_) of 20.93 μM (Figure 2).

### 2.2. Changes in Morphological Properties of HepG2 Cells Induced with Emodin

**Figure 3:**
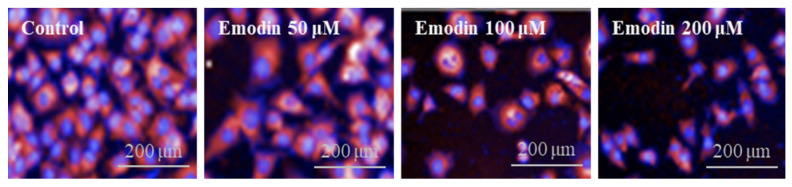
Morphological changes in HepG2 cells induced with emodin. Apoptotic nuclei appeared slightly smaller than nuclei in a normal state, and emodin treatment increased mitochondrial distribution. Hoechst 33342 and MitoTracker™ Deep Red were used to stain the nuclei and mitochondria, respectively.

A high-content screening (HCS) assay was used to investigate the effects of emodin on the morphological changes of HepG2 cells. The size of nuclear segmentation was measured using Hoechst channels, and the distribution and amounts of mitochondria were evaluated using MitoTracker™ Deep Red (Figure 3). To confirm the excessive morphological changes in HepG2 cells induced with emodin, cells were treated with relatively high concentrations of 25, 50, 100, and 200 μM. The changes in morphological properties observed included significant dose-dependent increases in cell area and cytoplasmic area values and, conversely, significant reductions in nuclear roundness and cytoplasmic intensity values (*p* < 0.05). In particular, the migration of mitochondria to the outer and inner nuclear regions after emodin treatment was observed by measuring the symmetry, threshold compactness, axial, or radial (STAR) descriptor, which can confirm the mitochondrial distribution. In addition, among the spots, edges, and ridges (SER) descriptors representing the mitochondrial texture, the values of seven descriptors excluding the SER edge were significantly increased, while the value of the SER edge was significantly decreased in a dose-dependent manner (Table 1). Thus, emodin treatment affected the viability of HepG2 cells and also induced morphological changes.

### 2.3. Changes in the Gene and Protein Expression of ER Stress and Apoptosis Markers Induced with Emodin

**Figure 4:**
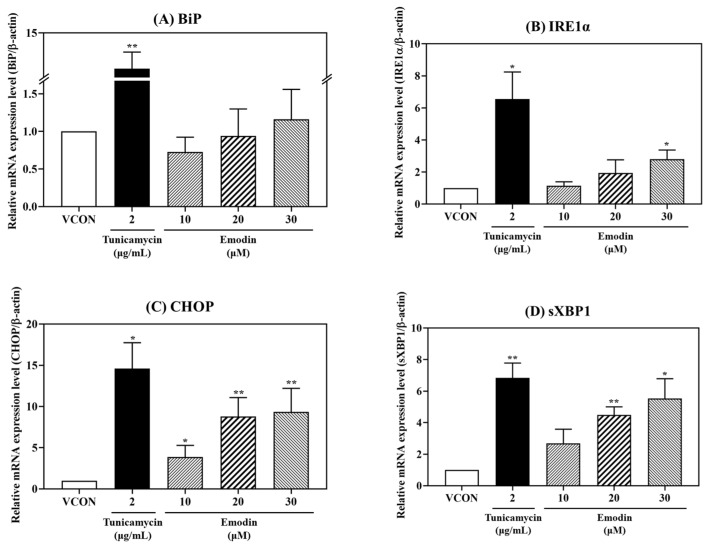
Relative mRNA expression levels of (**A**) BiP, (**B**) IRE1α, (**C**) CHOP, and (**D**) sXBP1 after treatment with emodin (10, 20, and 30 μM) for 24 h. The relative mRNA expression levels (**A**–**D**) increased after treatment with emodin. TM (2 μg/mL) served as the positive control. VCON, vehicle control; TM, tunicamycin. The data are shown as the mean ± SD from three independent experiments. * *p* < 0.05 and ** *p* < 0.01 (*t*-test) compared with the VCON group. The statistical power was ≥0.8, which means that a non-significant result is likely to be not really significant.

**Figure 5:**
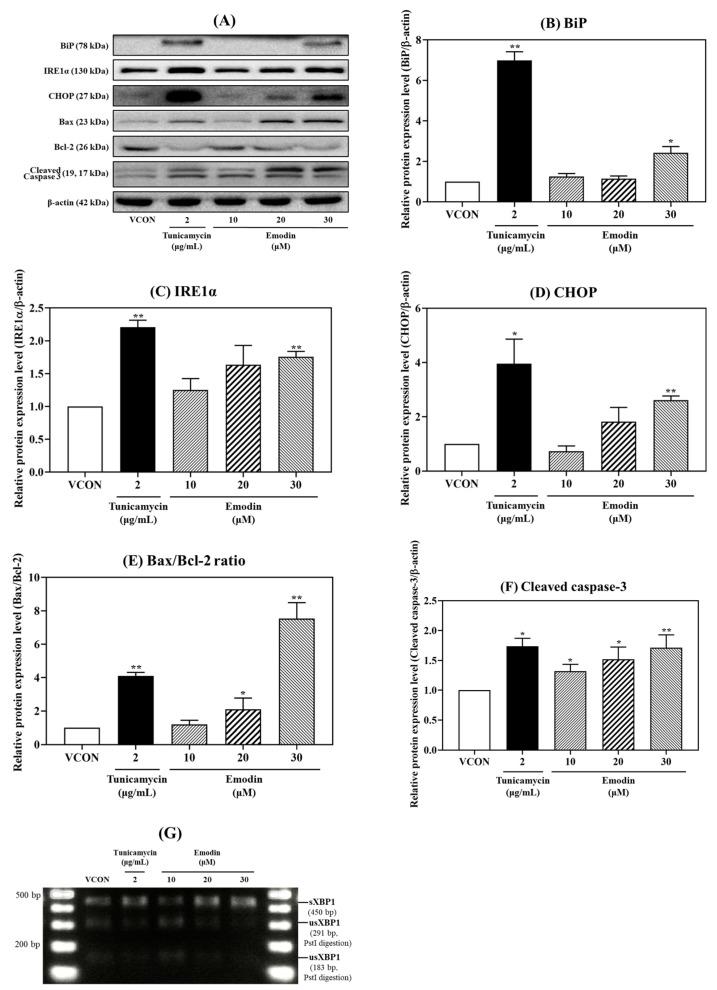
Relative protein expression levels of (**B**) BiP, (**C**) IRE1α, (**D**) CHOP, (**E**) Bax/Bcl-2 ratio, and (**F**) cleaved caspase-3 after treatment with emodin (10, 20, and 30 μM) for 24 h. (**A**) Relative protein expression levels of ER stress and apoptosis-related markers measured with a Western blot analysis. (**B**–**F**) The relative protein expression levels were increased with emodin treatment. (**G**) The effects of emodin on the expression of sXBP1 cut with *Pst*I. TM (2 μg/mL) served as the positive control. VCON, vehicle control; TM, tunicamycin. The data are shown as the mean ± SD from three independent experiments. * *p* < 0.05 and ** *p* < 0.01 (*t*-test) compared with the VCON group. The statistical power was ≥0.8, which means that a non-significant result is likely to be not really significant.

To investigate whether emodin induces ER stress and apoptosis, changes in the mRNA and protein levels of ER stress and apoptosis markers were determined with quantitative PCR (qPCR) and a Western blot analysis (Figure 4 and Figure 5), respectively. Tunicamycin (TM; 2 μg/mL) served as the positive control, and the mRNA levels of BiP, CHOP, IRE1α, and spliced XBP1 (sXBP1) were found to be affected by emodin treatment (Figure 4). Emodin tended to increase the relative mRNA expression level of BiP compared to the vehicle control, but the difference was not significant (Figure 4). The relative mRNA expression levels of CHOP, IRE1α, and sXBP1 were significantly increased with emodin treatment in a dose-dependent manner (Figure 4B–D). The relative protein expression of ER stress markers, such as BiP, IRE1α, and CHOP, was increased upon emodin treatment (Figure 5B–D); in particular, emodin increased IRE1α and CHOP relative protein expression in a dose-dependent manner (Figure 5C,D). Treating HepG2 cells with emodin also dose-dependently increased the Bax/Bcl-2 ratio and relative protein expression of cleaved caspase-3, which are related to apoptosis (Figure 5E,F).

To determine whether emodin induced the formation of sXBP1, cells were treated with the *Pst*I restriction enzyme to digest XBP1. Fragments digested with the *Pst*I restriction enzyme were evaluated to confirm whether emodin induces ER stress. After treating HepG2 cells with emodin, the expression of sXBP1 was dose-dependently increased, and remarkably, XBP1 digestion with *Pst*I was decreased (Figure 5G). Collectively, these data indicate that emodin can induce ER stress and apoptosis in HepG2 cells.

### 2.4. Evaluation of Apoptosis Induction Induced with Emodin Using Flow Cytometry

**Figure 6:**
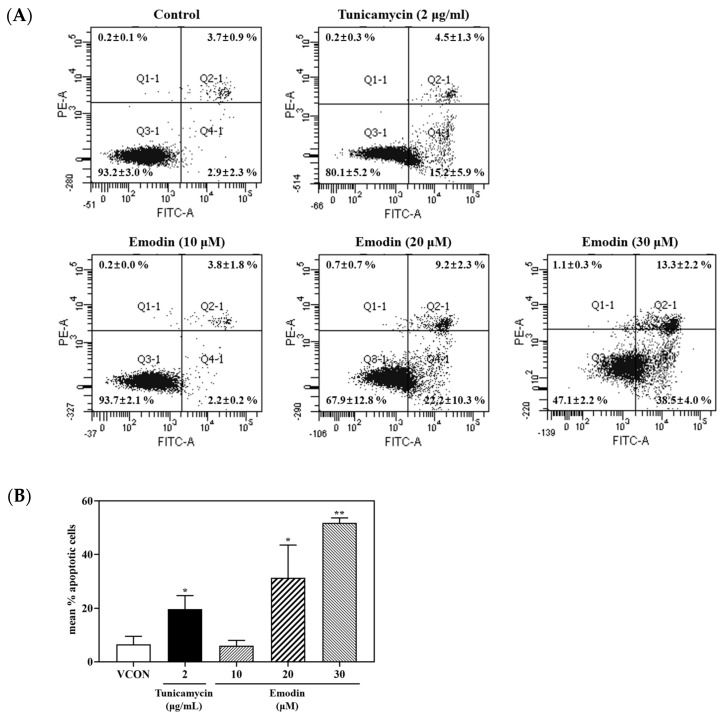
(**A**) Flow cytometry was used to measure the apoptosis rate using annexin V-FITC and PI double staining. (**B**) Treatment of HepG2 cells with emodin (10, 20, and 30 μM) increased the apoptosis rate in a dose-dependent manner. TM (2 μg/mL) served as the positive control. A total of 5000 events were collected per sample. VCON, vehicle control; TM, tunicamycin. The data are shown as the mean ± SD from three independent experiments. * *p* < 0.05 and ** *p* < 0.01 (*t*-test) compared with the VCON group. The statistical power was ≥0.8, which means that a non-significant result is likely to be not really significant.

Annexin V-fluorescein isothiocyanate (FITC) and propidium iodide (PI) were used to evaluate cell death and apoptosis, respectively. Cells were classified as necrotic (top left quadrant, Q1 in Figure 6A), late apoptosis (top right quadrant, Q2 in Figure 6A), live cells (bottom left quadrant, Q3 in Figure 6A), and early apoptosis (bottom right quadrant, Q4 in Figure 6A). As a result, emodin induced apoptosis in HepG2 cells in a dose-dependent manner (Figure 6B). These results indicate that emodin can induce apoptosis in HepG2 cells.

### 2.5. Changes in the Gene and Protein Expression of ER Stress and Apoptosis Markers Induced Using Cotreatment with Emodin and STF-083010

**Figure 7:**
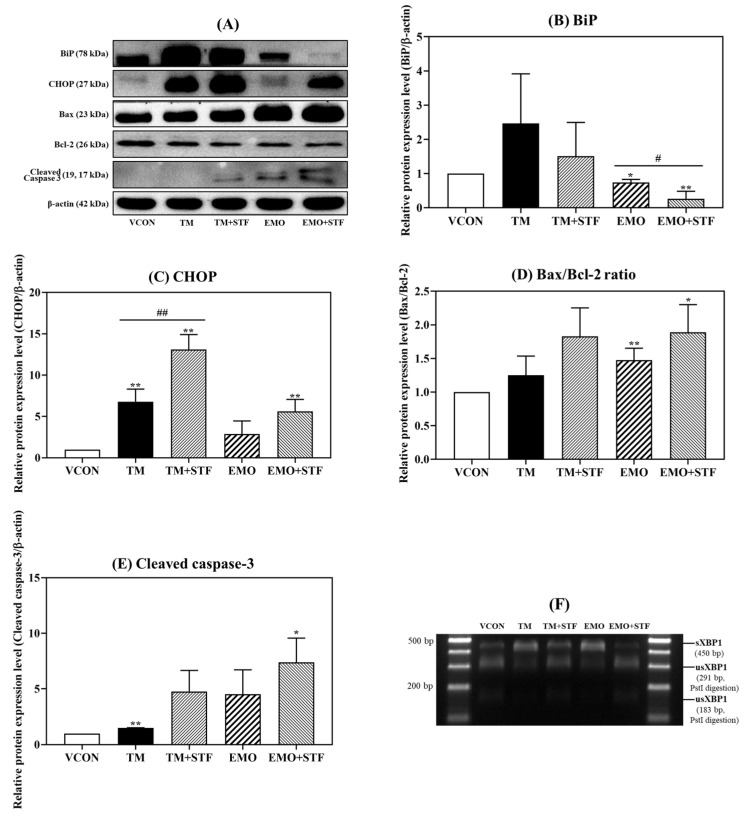
Relative protein expression levels of (**B**) BiP, (**C**) CHOP, (**D**) Bax/Bcl-2 ratio, and (**E**) cleaved caspase-3 after cotreatment with EMO (30 μM) and STF-083010 (100 μM) for 24 h. (**A**) Relative protein expression levels of ER stress and apoptosis-related markers measured with a Western blot analysis. The relative protein expression levels of (**B**) BiP were decreased and (**C**) CHOP, (**D**) Bax/Bcl-2 ratio, and (**E**) cleaved caspase-3 were increased compared with treatment with EMO alone. (**F**) The effects of EMO and STF-083010 on the expression of sXBP1 cut with *Pst*I. TM (2 μg/mL) served as the positive control. VCON, vehicle control; TM, tunicamycin; TM + STF, tunicamycin and STF-083010; EMO, emodin; EMO + STF, emodin and STF-083010. * *p* < 0.05 and ** *p* < 0.01 (*t*-test) compared with the VCON group. ^#^ *p* < 0.05 and ^##^ *p* < 0.01 (*t*-test) compared with the single treatment of TM or EMO. The statistical power was ≥0.8 (except BiP; 0.6), which means that a non-significant result is likely to be not really significant.

To elucidate whether emodin induces ER stress through the IRE1α–XBP1 pathway, cells were cotreated with STF-083010 (100 μM), a novel IRE1-XBP1 inhibitor, and emodin (30 μM). A Western blot analysis was conducted to evaluate the protein expression levels (Figure 7A), and TM (2 μg/mL) served as the positive control. Cells were also cotreated with TM and STF-083010 for comparison with emodin and STF-083010 cotreatment. The relative protein expression level of BiP was decreased upon cotreatment with STF-083010 and TM or emodin compared with the expression after single-agent treatments (Figure 7B). Conversely, the relative protein expression level of CHOP was increased after cotreatment with STF-083010 and TM or emodin compared with the expression after treatment with the agents alone (Figure 7C).

The relative protein expression of apoptosis-related markers was also increased using cotreatment with STF-083010 and TM or emodin. Cotreatment of HepG2 cells with emodin and STF-083010 increased the Bax/Bcl-2 ratio and relative protein expression level of cleaved caspase-3 compared with the expression level after treatment with emodin alone, similar to the effects after cotreatment with TM and STF-083010 (Figure 7D,E).

The formation of sXBP1 was also examined. Fragments digested with the *Pst*I restriction enzyme were evaluated, and the results confirmed that cotreatment with STF-083010 and TM or emodin reduced the formation of sXBP1 compared with sXPB1 formation induced with emodin or TM alone (Figure 7F).

## 3. Discussion

**Figure 8:**
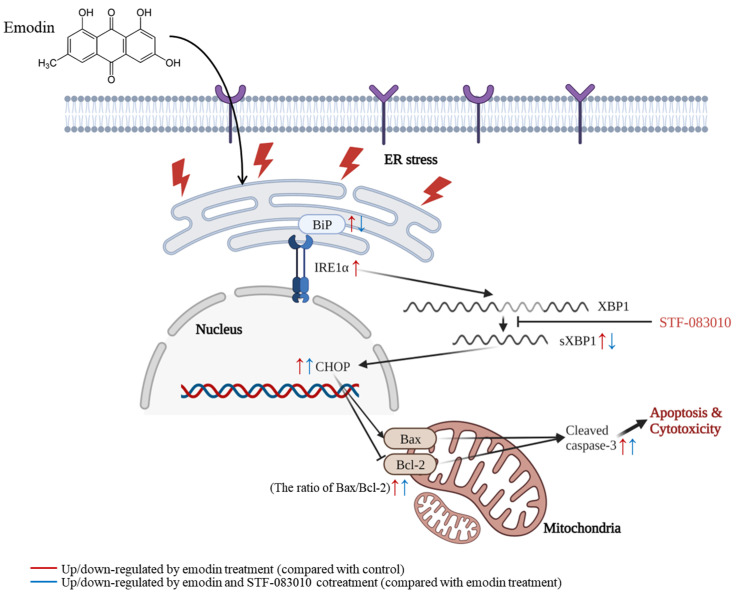
Overview of the possible molecular mechanism of ER stress and apoptosis induced with emodin in HepG2 cells. Created with BioRender.com.

The present study confirmed that emodin causes ER stress and apoptosis in human hepatocyte HepG2 cells and particularly causes ER stress through the IRE1α–XBP1 axis (Figure 8). Emodin decreased the metabolic activity of HepG2 cells and increased the relative protein expression of ER stress and apoptosis markers. Furthermore, emodin caused apoptosis in a dose-dependent manner, as confirmed with flow cytometry. To determine whether the IRE1α–XBP1 pathway is involved in emodin-induced ER stress, cells were cotreated with emodin and STF-083010, followed by measurement of the protein expression of ER stress and apoptosis markers. Compared with the relative protein expression levels induced by the single treatment with emodin, BiP was downregulated, and CHOP, the Bax/Bcl-2 ratio, and cleaved caspase-3 were upregulated.

Emodin is known as an emerging mycotoxin [2] and is a major component of some natural products, such as PMR [7]. Mycotoxins generated by the contamination of natural products with fungi can cause the total amount of emodin to increase. Thus, the toxicity of emodin should be evaluated. Exposure to emodin for 24 h decreased the metabolic activity of HepG2 cells (Figure 2). According to previous studies, emodin induces cytotoxic effects on HepG2 cells with IC_50_ values of 32.1 μM (MTT assay, after 24 h of treatment) [23] and 19.12 μM (Cell Counting Kit-8 assay, after 72 h of treatment) [24]. The IC_50_ value calculated in this study (20.93 μM) is generally consistent with previous studies.

Regarding the morphological changes induced with emodin, the cell area, cytoplasm area, compactness, and outer/inner membrane values were dose-dependently increased. In addition, the nucleus area, nucleus roundness, nucleus intensity, and cytoplasm intensity values were dose-dependently decreased. Distinct morphological changes appeared as the concentration increased, but most of the properties were not statistically significant at the 25 μM concentration of emodin (Table 1). Moreover, the number of cells at the same magnification decreased following treatment with emodin, and nuclei condensation and mitochondrial distribution occurred (Figure 3). HCS data numerically show that as apoptosis occurs, the nuclei of apoptotic cells appear somewhat smaller than nuclei in a normal state [25], and condensed and aggregated chromatin are observed as bright fluorescence due to DNA condensation [25].

A previous study reported that emodin could cause ER stress-related apoptosis through the activation of the BiP/IRE1α/CHOP signaling pathway in LO2 cells [20]. However, as LO2 cells are derivatives of the cervical cancer line HeLa, similar to Chang liver cells [22], it was necessary to investigate the hepatotoxicity of emodin using other hepatocytes. In this study, HepG2 cells were used as an in vitro liver model. HepG2 cells are used worldwide in pharmacological and toxicological research and are also known to be nontumorigenic cells with an epithelial-like morphology, a high proliferation rate, and the capability of performing liver functions [26]. Conversely, the expression of drug metabolites and transporters is restrained [26]. Thus, they were appropriately selected for the evaluation of hepatotoxicity induced with emodin, but it is possible that the data reported in this study may have been partially underestimated.

Treatment of HepG2 cells with emodin increased the relative mRNA expression (BiP, CHOP, IRE1α, and sXBP1) and relative protein expression (BiP, CHOP, IRE1α, Bax/Bcl-2 ratio, and cleaved caspase-3) of markers related to ER stress and apoptosis. For relative mRNA expression levels, only one reference gene (β-actin) was used for qPCR in this study. Thus, although it was recently reported that β-actin is one of the reference genes that can be used to normalize gene expression in HepG2 cells, the use of single reference genes has limitations in terms of qPCR accuracy [27]. Therefore, it is necessary to use appropriate combinations of three or more reference genes in future studies to improve qPCR accuracy. BiP, a central regulator of ER stress, controls the stasis between cell survival and apoptosis in ER stress-stimulated cells, especially through its interaction with caspases [28]. That emodin increased the relative protein expression of BiP in our study confirmed that emodin causes ER stress (Figure 5B). Among the three initiation phase mediators (PERK, IRE1, and ATF6), IRE1 is an ER transmembrane sensor that maintains ER and cell functions by activating the UPR. In particular, mammalian IRE1 promotes cell survival but causes apoptosis through the degradation of anti-apoptotic miRNA [13]. The treatment of HepG2 cells with emodin dose-dependently increased the relative protein expression level of IRE1α, showing that emodin induces ER stress through IRE1α activation (Figure 5C). In addition, activated IRE1α cleaves XBP1 mRNA, a substrate of IRE1 RNase, subsequently forming sXBP1 [29]. A specific region within unspliced XBP1 can be digested by the *Pst*I enzyme, which can be used to identify XBP1 that has not been cleaved by activated IRE1 [30]. In the current study, *Pst*I enzyme digestion was used to confirm that sXBP1 was increased and XBP1 was decreased after HepG2 cells were treated with emodin (Figure 5G). CHOP plays a pivotal role in apoptosis and mediates ER stress-induced apoptosis [31]. CHOP expression does not occur under non-ER stress conditions, but when ER stress occurs, its expression increases in IRE1-, PERK-, and ATF6-dependent manners [32]. That treatment with emodin dose-dependently increased the relative protein expression of CHOP in the current study confirmed that emodin could cause ER stress-induced apoptosis (Figure 5D).

In this study, the relative protein expression levels of Bcl-2, Bax, and cleaved caspase-3, all closely associated with apoptosis regulation, were measured after treatment with emodin. Apoptosis is regulated by the Bcl-2 family of genes. Bax promotes apoptosis, whereas Bcl-2 inhibits apoptosis [33]. Caspases are also important mediators of apoptosis, and caspase-3 activates the protease that induces apoptosis [34]. Therefore, caspase-3 is a major marker related to apoptosis and is activated upon its cleavage [35]. Data in the present study showed that the Bax/Bcl-2 ratio and the relative protein expression level of cleaved caspase-3 were increased after treatment with emodin (Figure 5E,F). Thus, emodin can induce apoptosis by activating ER stress, especially the IRE1α–XBP1 signaling pathway.

To investigate whether emodin induces ER stress through the IRE1α–XBP1 signaling pathway, cells were cotreated with STF-083010 and emodin, followed by the detection of the relative protein expression level of ER stress and apoptosis markers. STF-083010 is an IRE1 inhibitor that inhibits the generation of sXBP1 by suppressing IRE1 RNase activity [21]. Therefore, ER stress and apoptosis-related markers were measured to investigate how HepG2 cells were affected by cotreatment with emodin and STF-083010, which inhibited the IRE1–XBP1 pathway. The results showed that the relative protein expression level of BiP was decreased compared with that upon single emodin treatment, and the relative protein expression level of CHOP, the Bax/Bcl-2 ratio, and cleaved caspase-3 was increased compared with that using single emodin treatment (Figure 7B–E). These results showed the same tendency as the change in relative protein expression with single treatment of TM (positive control; induces ER stress by inhibiting *N*-glycosylation of proteins, thus accumulating misfolded protein [36,37]) and cotreatment with TM and STF-083010. The same tendency was also verified in a previous study using OVCAR-3 cells and SKOV-3 ovarian cancer cells regarding the protein expression of ER stress and apoptosis markers following cotreatment with TM and STF-083010 [38]. Interestingly, the relative protein expression levels of CHOP, the Bax/Bcl-2 ratio, and cleaved caspase-3 were increased using cotreatment with STF-083010 and TM. A previous study [38] suggested that the activation of PERK/ATF4 might upregulate CHOP because the PERK-ATF4 axis is the major pathway that activates CHOP. It has been shown that the activity of ATF4 is increased using cotreatment with STF-083010 and TM [38], which may explain the increased CHOP expression observed in our study. Furthermore, the Bax/Bcl-2 ratio and cleaved caspase-3, which are downstream proteins, were likely upregulated by the activation of CHOP.

Apoptosis induced using emodin treatment was measured with flow cytometry using annexin V-FITC and PI double staining. Emodin treatment dose-dependently increased apoptosis, indicating that emodin induces apoptosis (Figure 6B). These results support the increased relative protein expression levels of apoptosis-related markers induced with emodin treatment.

Collectively, this study investigated the effects of emodin on ER stress and apoptosis in HepG2 liver cells. Emodin led to cytotoxicity in a dose-dependent manner in HepG2 cells, suggesting that emodin may cause hepatotoxicity. The gene and relative protein expression levels of ER stress and apoptosis-related markers were upregulated with emodin treatment in a dose-dependent manner (except BiP); thus, we propose that emodin may cause ER stress and induce apoptosis. Additionally, changes in the gene and relative protein expression levels of ER stress and apoptosis-related markers after cotreatment of cells with STF-083010 and emodin demonstrated that emodin could cause ER stress through the IRE1α–XBP1 axis. Although the apoptosis-related marker expression was not statistically significantly reversed with inhibitor treatment, the relative protein expression level (CHOP, the ratio of Bax/Bcl-2, and cleaved caspase-3) tends to increase compared to single treatment with emodin. Thus, it can be suggested that apoptosis can be regulated through the IRE1α-XBP1 axis. To the best of our knowledge, this is the only research to use an IRE1α inhibitor to show that emodin causes ER stress by activating the IRE1α–XBP1 pathway. Therefore, our findings demonstrate that emodin can cause hepatotoxicity by inducing ER stress and apoptosis. However, this study was based on an in vitro cell model; thus, additional studies are needed in another in vitro or in vivo liver model. Furthermore, because emodin is an emerging mycotoxin, additional monitoring of food and feed is likely needed.

## 4. Materials and Methods

### 4.1. Chemicals and Reagents

Emodin (#E7881), TM (#T7765), MTT (#M2128), and dimethyl sulfoxide (DMSO) (#D8418) were purchased from Sigma-Aldrich (St. Louis, MO, USA). A specific IRE1α inhibitor, STF-083010 (#S7771), was obtained from Selleckchem (Houston, TX, USA), and the *Pst*I enzyme (#R019S) was purchased from Enzynomics, Inc. (Daejeon, Republic of Korea). A Dulbecco’s modified Eagle’s medium (DMEM; #11965-092), the materials used for the cell culture (e.g., penicillin), and phosphate-buffered saline (PBS) (#70011044) were obtained from Gibco Biotechnology (Rockville, MD, USA). A Thunderbird SYBR qPCR Mix (#QPS-201) was obtained from Toyobo (Osaka, Japan), and a radioimmunoprecipitation assay (RIPA) buffer was purchased from Rockland Immunochemicals (Limerick, Ireland). The primary antibodies against β-actin (#sc-47778), Bcl-2 (#sc-7382), and Bax (#sc-23959), and secondary antibodies (anti-rabbit, #sc-2357; anti-mouse, #sc-2005), were purchased from Santa Cruz Biotechnology (Dallas, TX, USA), and the primary antibodies against BiP (#3177S), CHOP (#2895S), IRE1α (#3294S), and cleaved caspase-3 (#9661S) were purchased from Cell Signaling Technology (Beverly, MA, USA). An electrochemiluminescence solution and buffers (e.g., gel-forming buffers, Tris/glycine/sodium dodecyl sulfate (SDS) buffer, and Tris/glycine buffer) were purchased from Bio-Rad (Hercules, CA, USA), and polyvinylidene difluoride (PVDF) membranes (#10600023) for the Western blot analysis were purchased from GE Healthcare (Amersham, UK).

### 4.2. Cells and Cell Culture

The HepG2 human hepatic cell line was obtained from the Korea Research Institute of Chemical Technology (Daejeon, South Korea). The cells were maintained in DMEM containing 10% (*v*/*v*) heat-inactivated fetal bovine serum and 1% (*w*/*v*) penicillin–streptomycin and incubated at 37 °C under a humidified atmosphere of 5% CO_2_.

### 4.3. Measurement of Cell Metabolic Activity

HepG2 cells were seeded in 96-well culture plates at a density of 2.0 × 10^4^ cells/well, and incubated for 24 h. Subsequently, cells were treated with 0, 1, 2.5, 5, 10, 15, 20, 30, 40, and 80 μM of emodin for 24 h. Emodin was dissolved in DMSO, and the final concentration of DMSO in the medium was maintained at 0.5% (*v*/*v*). Then, 20 μL of a 5 mg/mL MTT solution dissolved in PBS was added to each well, and the plate was incubated at 37 °C for 4 h. The supernatant was removed after the incubation, and insoluble formazan crystals were dissolved in DMSO. A ThermoMax microplate reader (Molecular Devices, San Jose, CA, USA) was used to measure the absorbance at 540 nm; 0.5% DMSO was used as a vehicle control, and the data are expressed relative to the control. The IC_50_ value was calculated using GraphPad Prism software (version 8.0.2; San Diego, CA, USA).

### 4.4. Detection of Morphological Properties (HCS Assay)

HepG2 cells were seeded in collagen-coated CellCarrier Ultra microplates (PerkinElmer, Waltham, MA, USA) at a density of 2.0 × 10^4^ cells/well, and incubated for 24 h. Subsequently, cells were treated with 25, 50, 100, and 200 μM of emodin for 24 h. Emodin was dissolved in DMSO, and the final concentration of DMSO in the medium was maintained at 0.5% (*v*/*v*). PBS was used to rinse the cells, and 4% paraformaldehyde (FUJIFILM Wako Pure Chemical Corporation, Osaka, Japan) was used for 20 min to fix the cells. Then, cells were washed twice with PBS and stained with DNA-specific fluorescent Hoechst 33342 (1.1 μM) and MitoTracker™ Deep Red (100 nM; Thermo Fisher Scientific, Waltham, MA, USA) for 30 min. An Operetta High-Content Imaging System (PerkinElmer) was used to observe the cells, and images were analyzed using Harmony software (PerkinElmer).

### 4.5. qPCR Analysis

HepG2 cells (80 × 10^4^ cells) were seeded in 60 mm culture plates for 24 h and treated with emodin (10, 20, and 30 μM) for another 24 h. The total RNA of cells was harvested using an RNeasy Kit (Qiagen, Hilden, Germany) following the manufacturer’s instructions. The RNA quality was verified with the 28S/18S ratio using agarose gel electrophoresis (Figure S3) as well as 260/230 and 260/280 nm absorbance ratios. Subsequently, the cDNA was synthesized by reverse-transcribing the RNA (1 μg) using a QuantiTect Reverse Transcription Kit (Qiagen). Fifty nanograms of cDNA, primers for the target genes, and a Thunderbird SYBR qPCR Mix were contained in the final PCR volume of 20 μL. Table S1 presents the sequences of the primers used. Gene expression was determined using a CFX96 Real-Time PCR System (Bio-Rad). The PCR conditions were as follows: initial denaturation at 95 °C for 3 min, followed by 40 cycles of denaturation at 95 °C for 15 s, annealing at 60 °C for 10 s, and extension at 72 °C for 30 s. The expression level of mRNA was analyzed using Bio-Rad CFX Manager software (Bio-Rad), and β-actin was used as the housekeeping reference gene for normalization. The SD and coefficient of variation (CV) of untransformed quantification cycle (Cq) values for β-actin in three independent experiments ranged from 0.4 to 0.8 and 2.1 to 3.9%, respectively. Melting curve analyses were performed to identify nonspecific PCR amplification (Figure S4). The ΔΔ*C*_T_ method was used to calculate gene expression, and the data are expressed relative to the vehicle control (0.5% DMSO).

### 4.6. XBP1 Splicing

PCR products derived from the XBP1 cDNA were digested with the *Pst*I restriction enzyme at 37 °C for 2 h. *Pst*I was inactivated by heating the mixture at 80 °C for 20 min after a 5 min cooling step. Mixtures of digested or nondigested PCR products and a STAR loading solution (Dyne Bio, Seongnam, Republic of Korea) were loaded onto 2.5% agarose gels and electrophoresed at 100 V for 1 h. The DNA fragments were visualized using the Gel Doc EZ Imager (Bio-Rad).

### 4.7. Western Blot Analysis

HepG2 cells (300 × 10^4^ cells) were seeded in a 100 mm culture plate for 24 h and treated with emodin (10, 20, and 30 μM) or cotreated with emodin (30 μM) and STF-083010 (100 μM) for another 24 h. The concentration of STF-083010 was selected as 100 μM from a preliminary experiment based on previous studies [39,40]. Cells were lysed in a RIPA buffer containing 1% protease inhibitor cocktail (Quartett, Berlin, Germany) and centrifuged at 12,000 rpm for 20 min to obtain protein. A bicinchoninic acid (BCA) protein assay was conducted to quantify the protein. Proteins (40 μg) were separated on 12% or 15% SDS–polyacrylamide gels and electrotransferred to a PVDF membrane using a SE22 transfer tank (Hoefer, Holliston, MA, USA). Membranes were blocked with 5% skim milk or 5% bovine serum albumin in Tris-buffered saline with Tween-20 (TBS-T) (Biosesang, Seongnam, Korea) at 4 °C for 2 h. Subsequently, the membranes were incubated with primary antibodies against BiP (1:1000), CHOP (1:1000), IRE1α (1:1000), Bax (1:1000), Bcl-2 (1:1000), cleaved caspase-3 (1:1000), and β-actin (1:1000) for 24 h at 4 °C. Then, the membranes were incubated with horseradish peroxidase-conjugated anti-mouse and anti-rabbit secondary antibodies (1:5000) at 4 °C for 2 h after washing the membrane with TBS-T. A Smart Chemi 500 Imager (Sage Creation, Beijing, China) and Lane ID software (version 4.0) were used to measure and quantify the protein expression, respectively.

### 4.8. Cell Apoptosis Analysis

An Annexin V-FITC/PI Apoptosis Detection Kit (BD Biosciences, Franklin Lakes, NJ, USA) was used to measure cell apoptosis according to the manufacturer’s protocol. Briefly, HepG2 cells (300 × 10^4^ cells) were seeded in a 100 mm culture plate for 24 h and treated with emodin (10, 20, and 30 μM) for another 24 h. After incubation, all cells were treated with trypsin, washed twice with cold PBS, and resuspended in a binding buffer. Then, the cells (10 × 10^4^) were stained with annexin V-FITC and PI for 15 min at 25 °C. The samples were analyzed using a FACS Aria II Cell Sorter (BD Biosciences), and 5000 events were used for each sample.

### 4.9. Statistical Analysis

The data from three independent experiments are expressed as the mean ± SD. Statistical analyses were performed using GraphPad Prism software (version 8.0.2) or SPSS statistical software (version 26.0) (Armonk, NY, USA). The data were analyzed using a Student’s *t*-test or one-way analysis of variance (ANOVA), followed by a Scheffe’s multiple range test. Statistical significance was set at *p* < 0.05. An acceptable statistical power was considered to be 0.8.

## Figures and Tables

**Table 1:** Alteration of phenotypic marker expression induced with emodin treatment.

Morphological Properties	Descriptor	VCON	Emodin 25 μM	Emodin 50 μM	Emodin 100 μM	Emodin 200 μM
Basic intensity and morphology	Cell area	1.00 ± 0.06 ^b^	1.02 ± 0.05 ^b^	1.02 ± 0.07 ^b^	1.35 ± 0.50 ^ab^	1.86 ± 0.94 ^a^
	Cell roundness	1.00 ± 0.01	1.00 ± 0.01	1.01 ± 0.01	1.01 ± 0.03	1.00 ± 0.04
	Nucleus area	1.00 ± 0.03 ^a^	0.99 ± 0.03 ^ab^	0.95 ± 0.03 ^abc^	0.94 ± 0.08 ^bc^	0.92 ± 0.05 ^c^
	Nucleus roundness	1.00 ± 0.01 ^a^	0.98 ± 0.01 ^ab^	0.96 ± 0.02 ^bc^	0.94 ± 0.04 ^cd^	0.92 ± 0.05 ^d^
	Nucleus intensity	1.00 ± 0.04 ^a^	0.91 ± 0.04 ^b^	0.87 ± 0.05 ^b^	0.86 ± 0.09 ^bc^	0.80 ± 0.06 ^c^
	Cytoplasm area	1.00 ± 0.07 ^b^	1.03 ± 0.07 ^b^	1.05 ± 0.09 ^b^	1.49 ± 0.68 ^ab^	2.18 ± 1.27 ^a^
	Cytoplasm intensity	1.00 ± 0.06 ^ab^	1.05 ± 0.04 ^a^	1.01 ± 0.06 ^ab^	0.89 ± 0.08 ^b^	0.68 ± 0.19 ^c^
STAR	Compactness, 60%	1.00 ± 0.03	1.02 ± 0.03	1.06 ± 0.03	1.03 ± 0.13	1.05 ± 0.08
	Radial	1.00 ± 0.03 ^b^	1.00 ± 0.02 ^b^	0.98 ± 0.03 ^b^	1.06 ± 0.09 ^ab^	1.12 ± 0.14 ^a^
	Juxtamembrane	1.00 ± 0.01 ^c^	1.01 ± 0.01 ^bc^	1.01 ± 0.02 ^bc^	1.03 ± 0.03 ^ab^	1.05 ± 0.04 ^a^
	Cytosolic	1.00 ± 0.00 ^a^	1.00 ± 0.00 ^a^	0.99 ± 0.01 ^a^	0.98 ± 0.01 ^b^	0.98 ± 0.01 ^b^
	Perinuclear	1.00 ± 0.01 ^a^	0.99 ± 0.01 ^ab^	0.97 ± 0.02 ^bc^	0.96 ± 0.02 ^cd^	0.93 ± 0.03 ^d^
	Outer nuclear	1.00 ± 0.02 ^c^	1.00 ± 0.02 ^c^	1.02 ± 0.03 ^bc^	1.07 ± 0.06 ^a^	1.05 ± 0.05 ^ab^
	Inner nuclear	1.00 ± 0.03 ^b^	1.02 ± 0.02 ^b^	1.05 ± 0.03 ^b^	1.13 ± 0.09 ^a^	1.12 ± 0.08 ^a^
SER	Spot	1.00 ± 0.03 ^b^	0.99 ± 0.02 ^b^	1.00 ± 0.03 ^b^	1.06 ± 0.04 ^b^	1.23 ± 0.15 ^a^
	Hole	1.00 ± 0.03 ^b^	0.99 ± 0.03 ^b^	1.01 ± 0.03 ^b^	1.06 ± 0.05 ^b^	1.24 ± 0.17 ^a^
	Edge	1.00 ± 0.02 ^a^	0.98 ± 0.02 ^ab^	0.96 ± 0.05 ^abc^	0.94 ± 0.05 ^bc^	0.93 ± 0.07 ^c^
	Ridge	1.00 ± 0.02 ^b^	0.99 ± 0.02 ^b^	0.99 ± 0.02 ^b^	1.02 ± 0.03 ^b^	1.14 ± 0.09 ^a^
	Valley	1.00 ± 0.02 ^b^	0.99 ± 0.02 ^b^	1.00 ± 0.03 ^b^	1.03 ± 0.03 ^b^	1.14 ± 0.10 ^a^
	Saddle	1.00 ± 0.02 ^b^	1.00 ± 0.02 ^b^	0.99 ± 0.02 ^b^	1.01 ± 0.02 ^b^	1.10 ± 0.06 ^a^
	Bright	1.00 ± 0.02 ^b^	0.99 ± 0.02 ^b^	1.00 ± 0.03 ^b^	1.04 ± 0.03 ^b^	1.18 ± 0.12 ^a^
	Dark	1.00 ± 0.02 ^b^	0.99 ± 0.02 ^b^	1.00 ± 0.03 ^b^	1.05 ± 0.04 ^b^	1.19 ± 0.13 ^a^

Data are expressed as the mean ± SD from three independent experiments. ^a–d^ Means in the same row without a common superscript letter differ (*p* < 0.05) as analyzed with one-way ANOVA, followed by Scheffe’s multiple range test. STAR: symmetry, threshold compactness, axial, or radial; SER: spots, edges, and ridges; VCON: vehicle control. The statistical power was ≥0.8 (except compactness; 0.4), which means that a non-significant result is likely to be not really significant.

## Data Availability

The data presented in this study are available on request from the corresponding author. Data sharing is not applicable to this article.

## Supplementary Materials

The following supporting information can be downloaded at: https://www.mdpi.com/article/10.3390/toxins15070455/s1, Table S1: List of primers used in qPCR, Figure S1: Original images of blot membranes in Figure 5(A), Figure S2: Original images of blot membranes in Figure 7(A), Figure S3: Agarose gel electrophoresis of all RNA samples used in qPCR analysis, Figure S4: Melting curves of qPCR amplification.

## Abbreviations

Annexin V-FITC: Annexin V-fluorescein isothiocyanate; ATF6: Activating transcription factor 6; Bax: Bcl-2-associated X protein; Bcl-2: B-cell lymphoma 2; BiP: Binding immunoglobulin protein; CHOP: C/EBP homologous protein; DMEM: Dulbecco’s modified Eagle’s medium; DMSO: Dimethyl sulfoxide; ER: Endoplasmic reticulum; IRE1α: Inositol-requiring enzyme 1 alpha; PBS: Phosphate-buffered saline; PERK: Protein kinase R-like endoplasmic reticulum kinase; PI: Propidium iodine; PMR: Polygoni Multiflori Radix; PVDF: Polyvinylidene difluoride; qPCR: Quantitative PCR; ROS: Reactive oxygen species; SD: Standard deviation; SER: Spots, edges, and ridges; STAR: Symmetry, threshold compactness, axial, or radial; sXBP1: spliced XBP1; TBS-T: Tris-buffered saline with Tween-20; TM: Tunicamycin; UPR: Unfolded protein response.

## References

[1] Gajęcki M.T., Gajęcka M., Zielonka Ł. (2020). The presence of mycotoxins in feed and their influence on animal health. Toxins.

[2] Wells J.M., Cole R.J., Kirksey J.W. (1975). Emodin, a toxic metabolite of *Aspergillus wentii* isolated from weevil-damaged chestnuts. Appl. Microbiol..

[3] Gruber-Dorninger C., Novak B., Nagl V., Berthiller F. (2017). Emerging mycotoxins: Beyond traditionally determined food contaminants. J. Agric. Food Chem..

[4] Sulyok M., Krska R., Schuhmacher R. (2010). Application of an LC–MS/MS based multi-mycotoxin method for the semi-quantitative determination of mycotoxins occurring in different types of food infected by moulds. Food Chem..

[5] Khoshal A.K., Novak B., Martin P.G.P., Jenkins T., Neves M., Schatzmayr G., Oswald I.P., Pinton P. (2019). Co-occurrence of DON and emerging mycotoxins in worldwide finished pig feed and their combined toxicity in intestinal cells. Toxins.

[6] Gallo A., Ghilardelli F., Atzori A.S., Zara S., Novak B., Faas J., Fancello F. (2021). Co-occurrence of regulated and emerging mycotoxins in corn silage: Relationships with fermentation quality and bacterial communities. Toxins.

[7] Na M.K., Park J.Y., An R.B., Lee S.M., Kim Y.H., Lee J.P., Seong R.S., Lee K.S., Bae K.H. (2000). Quality evaluation of Polygoni Multiflori Radix. Korean J. Pharmacogn..

[8] Dong X., Fu J., Yin X., Cao S., Li X., Lin L., Huyiligeqi, Ni J. (2016). Emodin: A review of its pharmacology, toxicity and pharmacokinetics. Phytother. Res..

[9] Xue J., Ding W., Liu Y. (2010). Anti-diabetic effects of emodin involved in the activation of PPARγ on high-fat diet-fed and low dose of streptozotocin-induced diabetic mice. Fitoterapia.

[10] Ma J., Zheng L., He Y.S., Li H.J. (2015). Hepatotoxic assessment of Polygoni Multiflori Radix extract and toxicokinetic study of stilbene glucoside and anthraquinones in rats. J. Ethnopharmacol..

[11] Lin L., Lin H., Zhang M., Ni B., Yin X., Qu C., Ni J. (2015). A novel method to analyze hepatotoxic components in *Polygonum multiflorum* using ultra-performance liquid chromatography-quadrupole time-of-flight mass spectrometry. J. Hazard. Mater..

[12] Liu X., Liu Y., Qu Y., Cheng M., Xiao H. (2015). Metabolomic profiling of emodin-induced cytotoxicity in human liver cells and mechanistic study. Toxicol. Res..

[13] Chen Y., Brandizzi F. (2013). IRE1: ER stress sensor and cell fate executor. Trends Cell Biol..

[14] Zeeshan H.M.A., Lee G.H., Kim H.R., Chae H.J. (2016). Endoplasmic reticulum stress and associated ROS. Int. J. Mol. Sci..

[15] Sano R., Reed J.C. (2013). ER stress-induced cell death mechanisms. Biochim. Biophys. Acta.

[16] Schröder M., Kaufman R.J. (2005). The mammalian unfolded protein response. Annu. Rev. Biochem..

[17] Szegezdi E., Logue S.E., Gorman A.M., Samali A. (2006). Mediators of endoplasmic reticulum stress-induced apoptosis. EMBO Rep..

[18] Saraste A., Pulkki K. (2000). Morphologic and biochemical hallmarks of apoptosis. Cardiovasc. Res..

[19] Elmore S. (2007). Apoptosis: A review of programmed cell death. Toxicol. Pathol..

[20] Qiu L.Z., Yue L.X., Ni Y.H., Zhou W., Huang C.S., Deng H.F., Wang N.N., Liu H., Liu X., Zhou Y.Q. (2021). Emodin-induced oxidative inhibition of mitochondrial function assists BiP/IRE1α/CHOP signaling-mediated ER-related apoptosis. Oxid. Med. Cell. Longev..

[21] Ming J., Ruan S., Wang M., Ye D., Fan N., Meng Q., Tian B., Huang T. (2015). A novel chemical, STF-083010, reverses tamoxifen-related drug resistance in breast cancer by inhibiting IRE1/XBP1. Oncotarget.

[22] Weiskirchen R. (2023). Letter to the Editor: LO2, a misidentified cell line: Some data should be interpreted with caution. Hepatology.

[23] Chen C., Gao J., Wang T.S., Guo C., Yan Y.J., Mao C.Y., Gu L.W., Yang Y., Li Z.F., Liu A. (2018). NMR-based metabolomic techniques identify the toxicity of emodin in HepG2 cells. Sci. Rep..

[24] Zhou R.S., Wang X.W., Sun Q.F., Ye Z.J., Liu J.W., Zhou D.H., Tang Y. (2019). Anticancer effects of emodin on HepG2 cell: Evidence from bioinformatic analysis. BioMed Res. Int..

[25] Doonan F., Cotter T.G. (2008). Morphological assessment of apoptosis. Methods.

[26] Donato M.T., Tolosa L., Gómez-Lechón M.J. (2015). Culture and functional characterization of human hepatoma HepG2 cells. Methods Mol. Biol..

[27] Gorji-Bahri G., Moradtabrizi N., Hashemi A. (2021). Uncovering the stability status of the reputed reference genes in breast and hepatic cancer cell lines. PLoS ONE.

[28] Lee A.S. (2005). The ER chaperone and signaling regulator GRP78/BiP as a monitor of endoplasmic reticulum stress. Methods.

[29] Kim R., Erni M., Tanabe K., Murakami S. (2006). Role of the unfolded protein response in cell death. Apoptosis.

[30] Kwon K., Goo T.W., Kwon O.Y. (2005). Development of rapid detection method for unfolded protein response in the mammalian cells. J. Exp. Biomed. Sci..

[31] Oyadomari S., Mori M. (2004). Roles of CHOP/GADD153 in endoplasmic reticulum stress. Cell Death Differ..

[32] Nishitoh H. (2012). CHOP is a multifunctional transcription factor in the ER stress response. J. Biochem..

[33] Brady H.J.M., Gil-Gómez G. (1998). Molecules in focus Bax. The pro-apoptotic Bcl-2 family member, Bax. Int. J. Biochem. Cell Biol..

[34] Porter A.G., Jänicke R.U. (1999). Emerging roles of caspase-3 in apoptosis. Cell Death Differ..

[35] Boatright K.M., Salvesen G.S. (2003). Mechanisms of caspase activation. Curr. Opin. Cell Biol..

[36] Guha P., Kaptan E., Gade P., Kalvakolanu D.V., Ahmed H. (2017). Tunicamycin induced endoplasmic reticulum stress promotes apoptosis of prostate cancer cells by activating mTORC1. Oncotarget.

[37] Banerjee A., Lang J.Y., Hung M.C., Sengupta K., Banerjee S.K., Baksi K., Banerjee D.K. (2011). Unfolded protein response is required in *nu/nu* mice microvasculature for treating breast tumor with tunicamycin. J. Biol. Chem..

[38] Barez S.R., Atar A.M., Aghaei M. (2020). Mechanism of inositol-requiring enzyme 1-alpha inhibition in endoplasmic reticulum stress and apoptosis in ovarian cancer cells. J. Cell Commun. Signal..

[39] Lei Z., Yang L., Yang Y., Yang J., Niu Z., Zhang X., Song Q., Lei Y., Wu H., Guo J. (2020). Activation of Wnt/β-catenin pathway causes insulin resistance and increases lipogenesis in HepG2 cells via regulation of endoplasmic reticulum stress. Biochem. Biophys. Res. Commun..

[40] Wang D., Hou C., Cao Y., Cheng Q., Zhang L., Li H., Feng L., Shen Y. (2018). XBP1 activation enhances MANF expression via binding to endoplasmic reticulum stress response elements within MANF promoter region in hepatitis B. Int. J. Biochem. Cell Biol..

