# Supplementary material for: Emodin, an Emerging Mycotoxin, Induces Endoplasmic Reticulum Stress-Related Hepatotoxicity through IRE1α–XBP1 Axis in HepG2 Cells
Source: Toxins (Basel). 2023 Jul 12;15(7):455. doi: 10.3390/toxins15070455 (PMC10467057; doi:10.3390/toxins15070455)
Supplement: Supplementary file 1 [file toxins-15-00455-s001.zip › toxins-2376600-supplementary.pdf]

Supplementary Materials: Emodin, an Emerging Mycotoxin, Induces Endoplasmic Reticulum Stress-Related Hepatotoxicity through IRE1α–XBP1 Axis in HepG2 Cells

Su Been Park, Gun Hee Cho, Young Eun Park and Hyang Sook Chun

Table S1. List of primers used in qPCR

**Gene**	**GenBank Accession number**	**Primer sequence**	**Product size (base pair)**	**Annealing temperatures of the primers**
BiP (GRP78)	X87949	5′-GGTGACCTGGTACTGCTTGATG-3′<br>5′-CCTTGGATTCAGTTTGGTCATG-3′	84bp	60 ℃
CHOP (GADD153)	S40706	5′-CTTGGCTGACTGAGGAGGAG-3′<br>5′-TCACCATTCGGTCAATCAGA-3′	312bp	60 ℃
sXBP1	NM_001394000	5′-CTGAGTCCGAATCAGGTGCAG -3′<br>5′-ATCCATGGGGAGATGTTCTGG-3′	59bp	60 ℃
IRE1	AF059198	5′-CTCAGAGACAGCGCGAGTAG-3′<br>5′-ATCTCAGCCTAGCTGTCCCA-3′	155bp	60 ℃
β-actin	HQ154074	5′-TCATCACCATTGGCAATGAG -3′<br>5′-CACTGTGTTGGCGTACAGGT-3′	154bp	60 ℃

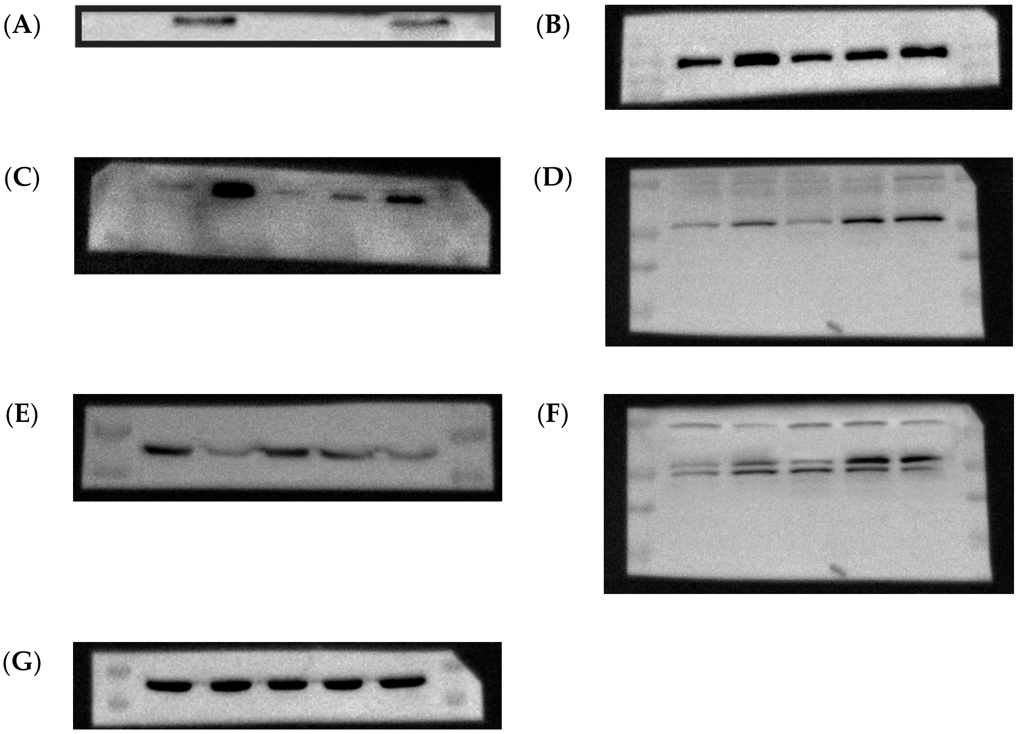

Figure S1. Original images of blot membranes in Figure 5(A). (A) Bip, (B) IRE1α, (C) CHOP, (D) Bax, (E) Bcl-2, (F) Cleaved caspase-3 and (G) β-actin.

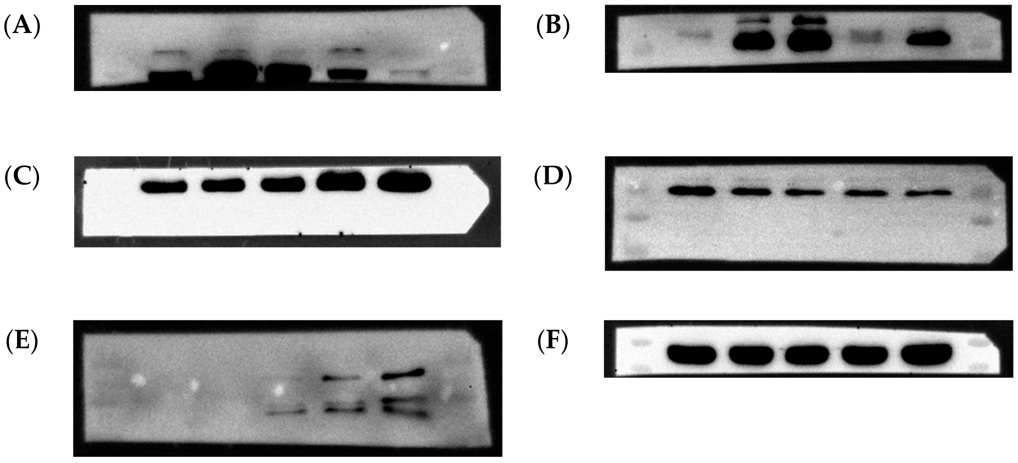

Figure S2. Original images of blot membranes in Figure 7(A). (A) Bip, (B) CHOP, (C) Bax, (D) Bcl-2, (E) Cleaved caspase-3 and (F) β-actin.

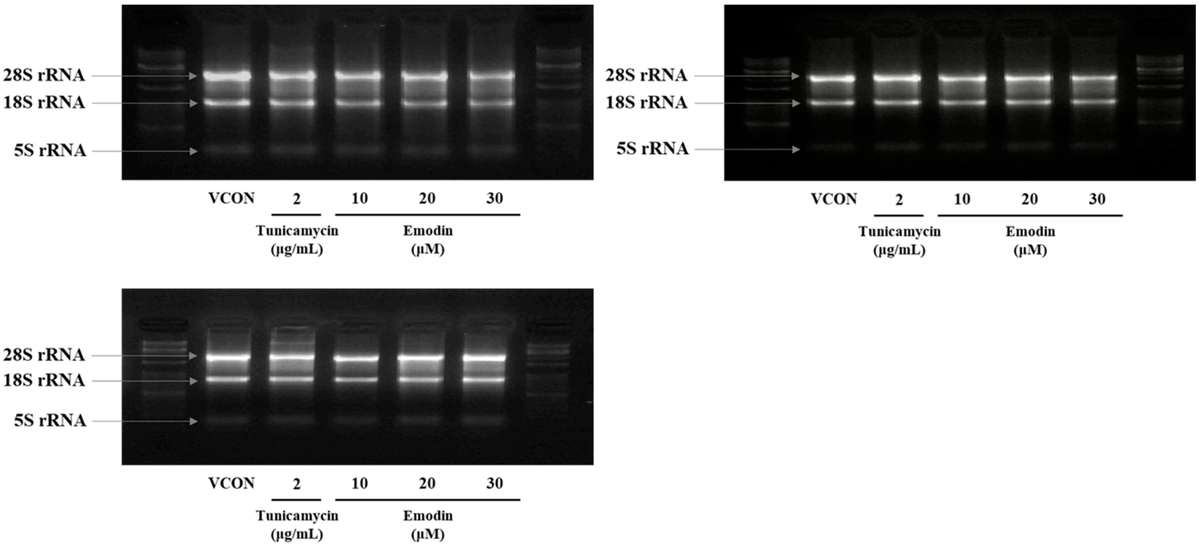

Figure S3. Agarose gel electrophoresis of all RNA samples used in qPCR analysis.

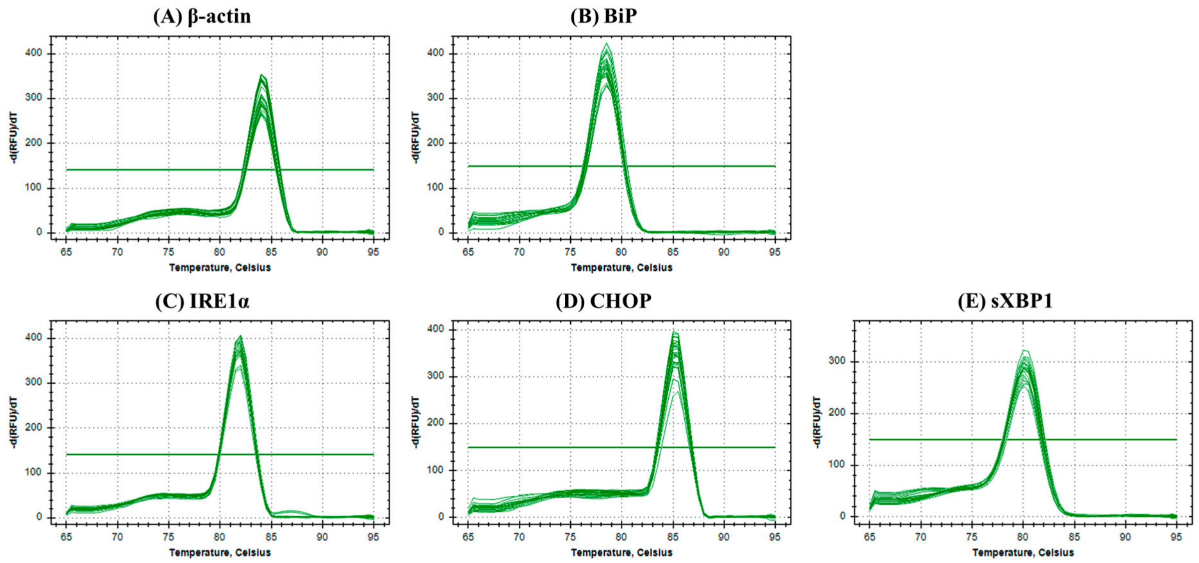

Figure S4. Melting curves of qPCR amplification. (A) β-actin, (B) BiP, (C) IRE1α, (D) CHOP and (E) sXBP1.
